# Multimorbidity and Polytherapy in Patients with Femoral Neck Fracture: A Retrospective Observational Study

**DOI:** 10.3390/jcm11216405

**Published:** 2022-10-29

**Authors:** Veronica Borsari, Francesca Veronesi, Elisa Carretta, Milena Fini

**Affiliations:** 1 Complex Structure Surgical Sciences and Technologies, IRCCS Istituto Ortopedico Rizzoli, Via Di Barbiano 1/10, 40136 Bologna, Italy; 2 Department of Programming and Monitoring, IRCCS Istituto Ortopedico Rizzoli, Via Di Barbiano 1/10, 40136 Bologna, Italy; 3 Scientific Direction, IRCCS Istituto Ortopedico Rizzoli, 40136 Bologna, Italy

**Keywords:** femoral neck fractures, multimorbidity, polytherapy, gender, retrospective study

## Abstract

Fractures of the femoral neck are one of the most common reasons for admission to an orthopedic institute. These patients also show multimorbidity (≥2 chronic conditions) and polytherapy (≥5 drugs). Multimorbidity and polytherapy are associated with a high risk of hospitalization and a reduction in quality of life. The present retrospective observational study was conducted to evaluate the prevalence of multimorbidity and polytherapy in patients aged ≥65 years and surgically treated for femoral neck fractures at an orthopedic institute over 3 years. Multimorbidity was evaluated with Elixhauser’s comorbidity measure and polytherapy was obtained from the patient’s medical record. This study identified 917 patients (84 ± 7.6 years); most of them were females. Most patients presented ≥2 chronic conditions, the most frequent of which was uncomplicated hypertension, and most patients used ≥5 drugs, of which antithrombotic ones were the most frequently taken. No significant gender and age differences were found between the presence or not of multimorbidity or polytherapy. Multimorbidity and polytherapy were statistically associated with an increased and decreased risk of 1-year mortality, respectively. This retrospective study has evaluated the variables required for the establishment of a minimum core of descriptors of the prevalence of polytherapy and multimorbidity in the orthopedic field.

## 1. Introduction

The aging of population is a constantly increasing phenomenon, with an associated rise in the prevalence of chronic diseases and in the number of drugs taken [1].

Italy is a country with a high seniority rate, with a citizenship over 65 that is constantly growing within the total number of the population [2]. These data indicate a higher life expectancy, both in terms of longevity and the general state of health and well-being necessary to reach an advanced age. According to Istituto Nazionale di Statistica (ISTAT), the average age of the Italian population is now 44.7 years, the highest ever recorded, and life expectancy at birth is 83.4 years [3].

Among the orthopedic diseases affecting the elderly population, the fractures of the femoral neck are the most common reasons for admission to an orthopedic institute, affecting more than 90,000 patients in Italy every year [4]. Proximal femoral neck fractures, excluding intertrochanteric ones, represent a disabling disease in 30% of cases with a significant increase in mortality [5].

Since most patients with femoral neck fractures are elderly, they also show other significant associated comorbidities, requiring a complex approach, with consequent high lengths of stay (LOS) in hospital, health costs for the national health system (NHS), morbidity and mortality (nearly 30% after 1 year) [6]. Femoral neck fractures are usually treated with a surgical approach, performed within 72 h upon arrival at the hospital, which is recommended in elderly patients [7,8]. In Italy, after the correct diagnosis, these patients are hospitalized and operated on within 48 h, as per the guidelines of the Ministry of Health [9].

Multimorbidity is defined as the simultaneous presence of two or more chronic conditions in the same patient, while polytherapy indicates the simultaneous use of several drugs. Multimorbidity and polytherapy, in elderly subjects, are associated with various negative health effects, including a high risk of hospitalization and a reduction in quality of life, and the onset of disability with the consequent increase in costs [10,11].

A disease is considered chronic if it has a prolonged duration, for at least 1 year, and (1) has left residual disability or deterioration in quality of life, or (2) has required a long period of treatment or rehabilitation. A consensus on definition and classification has been reached on clinical judgment for chronicity definition and for the classification into a manageable number of categories [12].

From a clinical point of view, polytherapy is defined based on the drug number used ≥5, or as the use of unnecessary or inappropriate drugs according to international criteria [13]. However, there are discrepancies in the literature on the concept of polytherapy, as some studies refer to lower thresholds (three or more drugs) or higher (up to ten or more drugs). It is undoubtedly a topic of extreme relevance in contemporary medicine, in particular in elderly patients in which it appears to be common in clinical practice.

Multimorbidity and polytherapy, if associated with frailty among the elderly population, contribute to a high clinical-care complexity, resulting in considerable management difficulties within NHS [5]. Frailty is defined as a syndrome with a high vulnerability to low-power stressors, with decreased functional reserve and resilience, multiorgan dysfunction or multimorbidity. Its prevalence usually increases with age [14], but it is also related to the presence of one or more diseases. Indeed, in a recent review, middle-aged patients, affected by different spine diseases, are considered frail [15]. Among diseases that can lead to frailty, the orthopedic ones are the main causes, with 50% of cases associated to lower limb fractures due to the loss of movement and independence [16].

The effects of polytherapy on public health have worsened during the COVID-19 pandemic when the geriatric population was severely affected, probably due to the presence of other co-morbidities, weak immunity, and concurrent administration of several drugs [17].

The aim of this retrospective observational study was to evaluate the prevalence of multimorbidity and polytherapy in patients aged ≥65 years treated for femoral neck fractures at IRCCS Istituto Ortopedico Rizzoli (IOR), using the Institute and administrative databases, such as hospital discharge reports (HDRs) and the patient’s medical record. This study was performed in the ambit of the AGING Network of Italian Research and Care Institutes (IRCCS). In addition, the current study aimed at evaluating the indispensable variables to constitute a minimum core of descriptors of the prevalence of polytherapy and multimorbidity in the orthopedic field. Until now, no literature study has yet described or investigated the prevalence of multimorbidity and/or drugs used in an orthopedic population cohort. The obtained results could represent a first step towards a large-scale evaluation to improve the quality of care for complex elderly patients.

## 2. Materials and Methods

This study was approved by Area Vasta Ethical Committee of Emilia Romagna region (CE-AVEC 927/2020/Oss/IOR). The present retrospective observational study included all patients admitted from 1 January 2017 to 31 December 2019 at IOR. Inclusion criteria were age ≥65 years; both males and females; residents in the Emilia-Romagna region; surgically treated at the IOR with a diagnosis of fracture of the femoral neck (ICD codes 9-CM 820.0–820.9); and no neoplastic diseases. Exclusion criteria were age <65 years; residents outside the Emilia-Romagna region; and the presence of neoplastic diseases.

Demographic information and clinical data of each patient, chronic conditions and hospitalization data were obtained from the Institute database and from the HDR. The information contained in the HDR were combined with the variables relating to drug therapy for chronic diseases present at the time of admission. In particular, the drug use was obtained from the patient’s medical record in the medical anamnesis and therapy sections. All information were collected and analyzed in pseudo-anonymous form using a unique code attributed to each patient included in the study. The collected data formed an electronic database (Excel), stored in a password protected computer known only by the experimenters.

All patients included in the study were identified with a numerical code, so that sensitive data were made anonymous and used in compliance with the privacy legislation in force.

### 2.1. Measures

Multimorbidity was assessed based on the Elixhauser’s comorbidity measure that identifies 30 comorbidities [18], and osteoporosis was also included because it is often related to the diagnosis of femoral neck fracture [19]. The chronic conditions were identified using ICD-9-CM diagnosis codes reported in HDR. In this study, the presence of each chronic condition and the number of comorbidities were determined for each patient. Multimorbidity was considered here as the simultaneous presence of two or more chronic conditions.

For polytherapy, the drug compounds (3rd level Anatomical Therapeutic Chemical-ATC code: pharmacological subgroup) were used to identify classes of drug. The drugs used were identified for each patient by the information reported in the patient’s medical record. Moreover, the number of drugs used was calculated, and the classification was ‘no polytherapy’ 0–4 drugs, ‘polytherapy’ ≥5 drugs, and ‘excessive polytherapy’ ≥10 drugs [20].

### 2.2. Statistical Analysis

Data were summarized by mean ± standard deviation for continuous variables, and by absolute frequency and percentage for categorical variables. Groups were compared using the chi-square or Fisher exact test, as appropriate, for categorical variables, or by the t-test or Wilcoxon–Mann–Whitney test, as appropriate, for continuous variables. Correlations between multimorbidity and polytherapy was assessed by the Spearman correlation coefficient (r). A logistic regression model with backward variable selection was used to calculate adjusted odds ratio (OR) and 95% confidence intervals (95%CI) for polytherapy in patients with multimorbidity compared to patients without multimorbidity. Mortality was defined as the time elapsed from the date of admission to the date of death. If death occurred more than one year after hospital discharge, the patient was censored. To identify the potential effect of multimorbidity and polytherapy on 1-year mortality, adjusted hazard ratio (HR) and 95%CIs were assessed using the Cox regression model. Age, sex and length of stay were included in the multivariate model for both polytherapy and mortality outcomes. All tests were two sided with a significance level of 0.05. Analyses were performed using SAS 9.4 (SAS Institute, Cary, NC, USA) software.

## 3. Results

### 3.1. Demographic Data

In Table 1, the basal demographic data of the patients are summarized. We evaluated 917 patients with a mean age of 84 ± 7.6 years, (range 65–104). Most patients (*n* = 710, 77.5%) were between 65 and 90 years old, while four patients were over 100 years old (0.4%). Most patients were females (*n* = 706, 77.0%), while there were 211 males (23.0%). Regarding education, most patients declared that they have no academic qualifications (*n* = 430, 46.9%), while six patients had a bachelor’s degree (*n* = 6, 0.7%). Inside the region, most patients were resident in Bologna (*n* = 906, 98.8%).

Regarding hospitalization, almost all patients had emergency hospital admission (*n* = 916, 99.9%) with a mean LOS of 9.6 ± 4.1 days. Most patients were discharged at home (*n* = 735; 80.1%), 49.1% of these had the activation of the integrated home assistance service (361/735), and 18.9% (*n* = 173) of patients were transferred to other institutions such as acute care or rehabilitation facilities. Nine patients died during hospitalization (1.0%).

### 3.2. Multimorbidity

**Figure 1:**
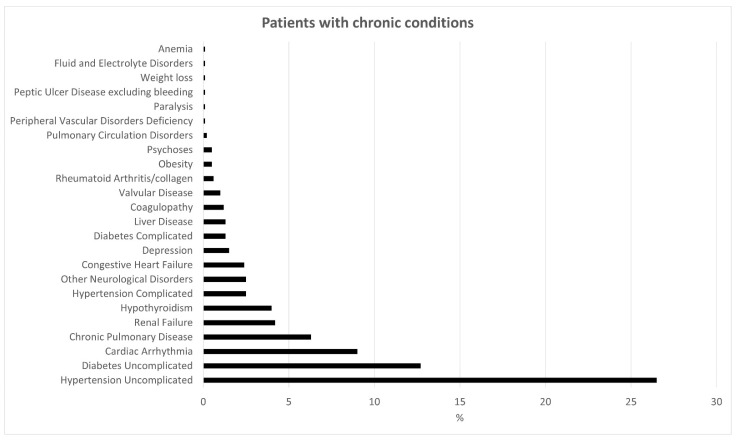
Percentage distribution (%) of the chronic conditions in the study population.

Figure 1 summarizes all the chronic conditions identified in the 917 patients of the cohort, employing the Elixhauser index. Seven conditions were not considered because AIDS, blood loss anemia, alcohol abuse and drug abuse were not present, while patients with lymphoma, metastatic cancer and solid tumor without metastasis were excluded at the beginning of the studies. Among the 917 patients, 464 (50.6%) had chronic conditions, while 453 patients (49.4%) had none. The most frequent pathologies were uncomplicated hypertension in 243 patients (26.5%), uncomplicated diabetes in 117 patients (12.7%) and cardiac arrhythmia in 83 patients (9.1%), while the least present conditions were the peripheral vascular disorders, paralysis, peptic ulcer disease excluding bleeding, weight loss, fluid and electrolyte disorders and anemia in one patient each (0.11%) (shown in Figure 1).

In our cohort, 184 patients (20.1%) presented multimorbidity (≥2 comorbid conditions). Among the patients that showed chronic conditions, 280 (30.5%), 118 (12.9%), 48 (5.2%), 16 (1.7%) and 2 (0.22%) patients had 1, 2, 3, 4 and 6 comorbidities together, respectively (Table 2).

No significant gender and age differences were found between patients with multimorbidity (≥2 comorbid conditions) and without multimorbidity (<2 comorbid conditions), (*p* = 0.647 and *p* = 0.280).

### 3.3. Polytherapy

**Figure 2:**
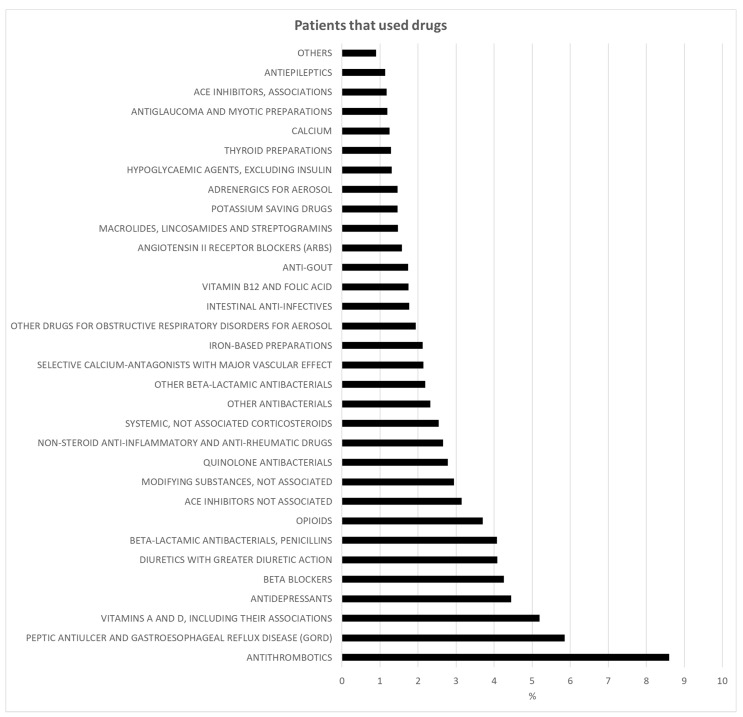
Percentage distribution (%) of the different drug types taken by the study population.

Based on the 3rd level ATC code, IOR patients took up to 29 drugs. Most patients took antithrombotic drugs (8.6%), drugs for peptic ulcer and gastroesophageal reflux disease (5.9%), and vitamins A and D (5.19%) (shown in Figure 2). Antidepressants were taken by 4.5% of the patients, beta-blocking agents by 4.3%, diuretics by 4.1%, opioids by 3.7%, and calcium channel blockers by 2.1%. Most patients usually used five or more drugs (*n* = 815; 88.9%) (Table 2), without a difference between females and males (89.5% vs. 86.7%, *p* = 0.258). No difference in the mean age of the patients was observed between polytherapy (≥5 drugs) and no polytherapy patients (83 ± 8.7 vs. 83.6 ± 7.4, *p* = 0.576).

**Figure 3:**
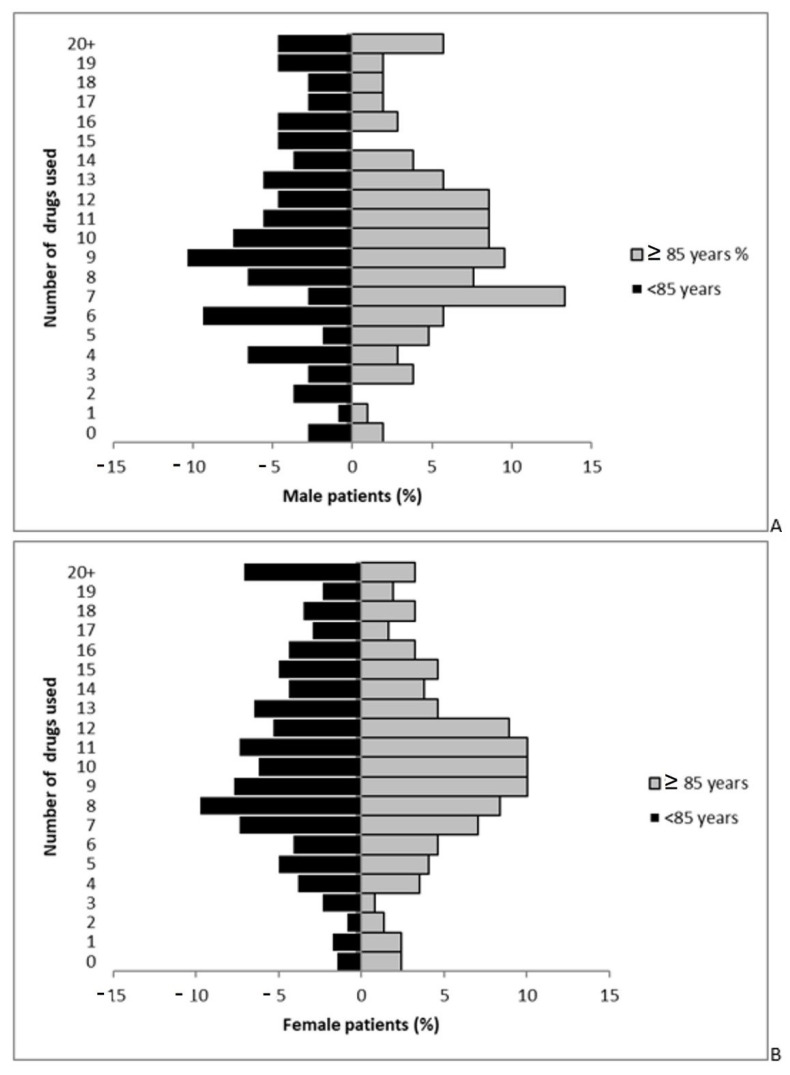
Pyramid charts of: (**A**) drug use in male patients by age; (**B**) drug use in female patients by age.

Overall, as observed in Figure 3, the drugs used had a similar profile between patients <85 years and patients ≥85 years both in males and females.

A weak positive correlation was assessed within the number of drugs used and the number of chronic conditions (r = 0.3, *p* < 0.0001). In multivariable analysis, patients with ≥2 comorbid conditions had a statistically increased risk of polytherapy defined as taking ≥5 drugs, compared to patients without multimorbidity (OR = 3.0; 95%CI: 1.4–6.4, *p* = 0.004) (Table 3). In addition, polytherapy statistically increased LOS by more than nine days (OR = 1.8; 95%CI: 1.1–2.8, *p* = 0.015) (Table 3).

One-year mortality after surgery was 18.5% (*n* = 170). The presence of comorbid conditions was statistically associated with an increased risk of 1-year mortality (HR = 1.7; 95%CI: 1.2–2.4, *p* = 0.003), whereas polytherapy was statistically associated with a decreased risk of 1-year mortality (HR = 0.3; 95%CI: 0.2–0.5, *p* < 0.0001) (Table 4).

## 4. Discussion

The results of this study indicate that multimorbidity affected 20.1% of patients aged ≥65 years treated for femoral neck fractures at IOR Institute over 3 years, while most of the patients underwent polytherapy, using 5 or more drugs (88.9%). 

In 2018, Italians over 65 years numbered 13.8 million, growing over time, and in the ten years from 2009 to 2019, the total number of centenarians went from 11,000 to over 14,000, with 84% of them women [21]. The increase in the average age is often accompanied by an increase in chronic conditions and in the number of drugs taken.

In this study, 917 patients affected by femoral neck fractures and with a mean age of 84 ± 7.6 years old have been evaluated. Most patients were females (77.0%). This is in line with the literature data because femoral neck fractures are the most frequent among all osteoporotic-related fractures of the hip (40–50%) and are three times more frequent in women than in men [22]. All patients arrived at IOR in the emergency room, since femoral neck fracture is an acute trauma and should be usually operated on within 24–48 h [23].

Concerning multimorbidity, the present study showed that 50.6% of the study population had at least one chronic condition, and that 20.1% of them had two or more chronic conditions.

Multimorbidity has an increasing relevance on epidemiology and economic impact on the NHS, representing one of the main challenges and increasing the risk of clinical fragility fractures [24].

While a consensus has been reached on the definition of multimorbidity as “the simultaneous presence of two or more chronic diseases”, no standard exists regarding its measurement [12]. Epidemiologic studies have demonstrated that multimorbidity is associated with an increased risk of death, disability, poor functional status, poor quality of life, adverse drug events and other adverse outcomes [25]. Thus, it becomes important to summarize the multimorbidities in a single score, but great heterogeneity exists in the prevalence estimation due to methodological differences used to determine chronic conditions and the source population.

The two most employed indexes are the Charlson comorbidity index and the Elixhauser comorbidity measure. The present study employed the latter, which identified 30 comorbidities, 17 of which were in common with the Charlson comorbidity measure, and it has a major impact on the short-term outcomes in acute hospital patients [18]. Osteoporosis was included among the considered chronic conditions because it is often related to the diagnosis of femoral neck fracture [19]

The results of a literature study on women with hip fractures indicated that greater comorbidity burden, estimated by the Elixhauser method, was associated with higher total health care costs after hip fracture [26]; moreover, a multicenter prospective study, conducted in men ≥ 65 years with osteoporotic hip fracture, showed that multimorbidity was associated with a high risk of hip fractures [27].

Similarly to our study, Fortuna D. et al. recently conducted an observational study based on regional healthcare data record linkage. These authors showed a prevalence of 61% of people ≥65 years resident in the Emilia-Romagna region in 2018 who suffered from ≥2 concurrent chronic diseases [28]. These data may appear higher than ours, but we must consider that we used a cohort of patients with femoral neck fractures operated at the IOR and not the entire population of the region. In addition, the number of diseases included in the multimorbidity definition can affect the prevalence rate in each different study.

Among the different chronic conditions present in the cohort of patients of the present study, hypertension was the most associated with femoral neck fractures (26.5%), followed by diabetes, heart and lung diseases. In the study by Fortuna D. et al., four groups of chronic condition that prevalently coexisted regarded cardiovascular, neuropsychiatric, metabolic, and pain patterns [28].

The association between fracture risk and hypertension was also observed by Li C. et al., asserting that hypertension is usually accompanied by reduced bone mineral density [29]. In a recent review on the epidemiology of hip fractures in Malaysia, it was found that 77.7% of individuals had at least one comorbid illness, and 26% were considered multimorbid. The most common chronic conditions were diabetes mellitus with a prevalence between 25–46%, hypertension between 34–74% and ischemic heart disease between 3–11%. These data are coherent with the results of our study. However, only bone-related medications were reported in 12% of patients and no information on polytherapy was provided [30].

As regards to polytherapy, in the present study most patients usually used ≥5 drugs (88.9%). The factors that determine the prescription of a large and growing number of drugs in elderly patients over the last few years are essentially three: (1) the availability of an increasing number of drugs for the treatment or prevention of specific chronic diseases (e.g., hypertension, diabetes, heart failure, etc.), that implies that the therapies recommended by the guidelines increasingly include numerous drugs (most current guidelines do not explicitly refer to the coexistence of other chronic diseases or to long-term exposure to a large number of drugs); (2) multimorbidity; (3) the fragmentation of care, which is why most patients receive prescriptions from multiple doctors [24]. A retrospective cohort study, performed on patients ≥70 years and with hip or proximal humerus fractures, showed that most patients were women, and the prescriptions of potentially inappropriate medication in older trauma patients increased adverse drug reactions and fractures [31].

Regarding the type of drugs taken, in our cohort of patients the antithrombotic ones were the most used. Since 2009, there has been a consensus statement that recommends the use of antithrombotic agents in hip and knee prothesis and in the treatment of femoral neck fractures to prevent venous thromboembolism [32].

In a recent retrospective study, conducted in a Swedish cohort of patients aged ≥75 years with hip fractures, antithrombotic drugs were not investigated, but it was observed that opioids, dopaminergic agents, anxiolytics, antidepressants and hypnotics/sedatives increased hip fractures, while vasodilators, antihypertensive agents, diuretics, beta-blocking agents, calcium channel blockers and renin–angiotensin system inhibitors were not associated with these fractures [33].

The present retrospective study found a positive correlation between the number of drugs taken and the number of comorbidities present, suggesting that the presence of at least one chronic condition is sufficient to determine a risk of polytherapy.

It was observed that multimorbidity (>2 chronic conditions) significantly increased the risk of death 1 year after surgery, while polytherapy (>5 drugs) significantly reduced this risk, adjusted for age and gender. The latter result seems consistent with the fact that adequately treated patients have a lower risk of death at 1 year.

The present study showed no significant gender and age differences in multimorbidity and polytherapy. While some studies observed different multimorbidity patterns between females and males, a recent secondary analysis study showed that hypertension, the most common comorbidity found in the present study, was the most common chronic disease in both females and males without gender differences in patients aged ≥65 years [34].

The limitations of the present work are represented by (i) possible omissions in medical records or the prescription of un-recorded off-label drugs, and (ii) the exclusion of patients with neoplastic diseases that represent 14.2% of people with an age ≥65 years in the Emilia-Romagna region [28] in order to analyze only fragility fractures. These limitations may have led to an underestimation of polytherapy and multimorbidity. Moreover, patients resident outside the Emilia-Romagna region were excluded because no access to databases on mortality from other regions was available.

## 5. Conclusions

The prevalence of multimorbidity and polytherapy in patients aged ≥65 years and surgically treated for femoral neck fractures at an orthopedic institute over 3 years were, respectively, 20.1% and 88.9%, with a positive correlation between the number of drugs taken and the number of comorbidities present. This retrospective study sets the stage for the evaluation of the variables required for the establishment of a minimum core of descriptors of the prevalence of polytherapy and multimorbidity in the orthopedic field.

In the literature, no other study has evaluated the prevalence of multimorbidity and polytherapy in a cohort of patients operated on for femoral neck fractures, associating multimorbidity and polytherapy and the risk of death. Most of the literature studies evaluated multimorbidity and polytherapy in a large cohort of the elderly population in general, without including a specific medical discipline such as orthopedics or traumatology.

The analysis of multimorbidity and polytherapy provides essential information to support health policy in the field of prevention, clinical management and resource allocation to guarantee personalized and adequate strategies for patients with multiple chronic pathologies.

## Figures and Tables

**Table 1:** Basal demographic data of the study population.

	All Sample (*n* = 917)
**Sex, *n* (%)**	
Female	706 (77.0)
Male	211 (23.0)
**Age (years), mean ± std**	84 ± 7.6
**Age, *n* (%)**	
65–69	51 (5.6)
70–74	74 (8.1)
75–79	131 (14.3)
80–84	187 (20.4)
85–89	267 (29.1)
90–94	168 (18.3)
95–99	35 (3.8)
>100	4 (0.4)
**Education, *n* (%)**	
No academic qualifications	430 (46.9)
Primary school diploma	276 (30.1)
Middle school graduation	117 (12.8)
High school graduation	78 (8.5)
University degree	10 (1.1)
Bachelor’s degree	6 (0.7)

**Table 2:** Multimorbidity and polytherapy in the study population.

	All Sample (*n* = 917)
**Number of chronic conditions, *n* (%)**	
0	453 (49.4)
1	280 (30.5)
2	118 (12.9)
3	48 (5.2)
4	16 (1.7)
6	2 (0.2)
**Number of drugs used, *n* (%)**	
0	19 (2.1)
1–4	83 (9.0)
5–9	317 (34.6)
≥10	498 (54.3)

**Table 3:** Results from multivariable logistic model for polytherapy. OR = odds ratio; LOS = length of stay in hospital.

Variable		OR (95%CI)	*p*-Value
Multimorbidity (≥2 comorbid conditions)	Yes vs. No	3.0 (1.4–6.3)	0.004
LOS (days)	>9 vs. ≤9	1.8 (1.1–2.8)	0.013

**Table 4:** Results from multivariable Cox model for 1-year mortality. HR = Hazard ratio; LOS = length of stay in hospital.

Variable		HR (95%CI)	*p*-Value
Polytherapy (≥5 drugs)	Yes vs. No	0.3 (0.2–0.5)	<0.0001
Multimorbidity (≥2 comorbid conditions)	Yes vs. No	1.7 (1.2–2.4)	0.003
Sex	M vs. F	2.6 (1.9–3.5)	<0.0001
Age (years)	≥85 vs. <85	2.9 (2.0–4.0)	<0.0001
LOS (days)	>9 vs. ≤9	1.1 (0.8–1.5)	0.531

## Data Availability

The raw data supporting the conclusions of this article will be made available by the authors, upon reasonable request, without undue reservation.
